# Supplementary material for: The Sustainability of Sweet Potato Residues from Starch Processing By-Products: Preparation with Lacticaseibacillus rhamnosus and Pediococcus pentosaceus, Characterization, and Application
Source: Foods. 2022 Dec 27;12(1):128. doi: 10.3390/foods12010128 (PMC9818312; doi:10.3390/foods12010128)
Supplement: Supplementary file 1 [file foods-12-00128-s001.zip › foods-2037440-supplementary-final.pdf]

Table S1 Total and free amino acids of sweet potato residues fermented with combined strains

	Total amino acids (mg/100 g DW)		Free amino acids (mg/100 g DW)	
	**SPR**	**FSPR**	**SPR**	**FSPR**
Asp$^{\Delta}$	0.51±0.05	0.46±0.02	0.11±0.01	0.12±0.06
Thr′	0.18±0.01	0.19±0.01	0.07±0.01	0.07±0.01
Ser	0.18±0.01	0.18±0.00	0.06±0.00	0.07±0.01
Glu$^{\Delta}$	0.32±0.02	0.30±0.02	0.17±0.00	0.10±0.01
Gly$^{\Delta}$	0.15±0.01	0.14±0.00	0.03±0.00	0.04±0.01
Ala$^{\Delta}$	0.17±0.01	0.18±0.01	0.11±0.03	0.08±0.02
Cys	0.00±0.00	0.00±0.00	0.01±0.00	0.01±0.00
Val′	0.19±0.03	0.18±0.00	0.07±0.01	0.06±0.01
Met′	0.01±0.01	0.01±0.01	0.01±0.00	0.01±0.00
Ile′	0.16±0.02	0.14±0.02	0.00±0.00	0.00±0.00
Leu′	0.24±0.04	0.21±0.02	0.08±0.01	0.08±0.02
Tyr	0.15±0.04	0.10±0.05	0.03±0.00	0.04±0.00
Phe′	0.19±0.01	0.15±0.01	0.08±0.03	0.06±0.00
Lys′	0.20±0.00	0.18±0.01	0.06±0.00	0.06±0.01
His′	0.07±0.02	0.07±0.01	0.02±0.00	0.03±0.01
Arg	0.12±0.09	0.17±0.02	0.04±0.00	0.07±0.01
Pro	0.10±0.03	0.13±0.01	0.06±0.00	0.06±0.01
∑AA	2.94±0.03	2.78±0.12	0.98±0.07	0.94±0.06
FAA$^{\Delta}$	1.16±0.09	1.08±0.06	0.40±0.04	0.35±0.04
EAA′	1.23±0.05	1.12±0.07	0.38±0.03	0.37±0.02
NEAA	1.70±0.02	1.66±0.05	0.63±0.04	0.59±0.09
EAA/∑AA	41.84±1.23	40.36±0.80	38.76±0.11	39.07±4.75

SPR: Sweet potato residues; FSPR: Sweet potato residues fermented under optimal process. Δ and ′: indicating flavor amino acids and essential amino acids, respectively; ∑AA: total amino acids; FAA$^{\Delta}$: total flavor amino acids; EAA′: total essential amino acids; NEAA: total non-essential amino acids.
